# The nephrogenic potential of the transcription factors osr1, osr2, hnf1b, lhx1 and pax8 assessed in Xenopus animal caps

**DOI:** 10.1186/1471-213X-11-5

**Published:** 2011-01-31

**Authors:** Christiane Drews, Sabine Senkel, Gerhart U Ryffel

**Affiliations:** 1 Institut für Zellbiologie (Tumorforschung) Universitätsklinikum Essen, Universität Duisburg-Essen, Hufelandstrasse 55, 45122 Essen, Germany

## Abstract

**Background:**

The three distinct types of kidneys, pronephros, mesonephros and metanephros, develop consecutively in vertebrates. The earliest form of embryonic kidney, the pronephros, is derived from intermediate mesoderm and the first expressed genes localized in the pronephros anlage are the transcription factors osr1, osr2, hnf1b, lhx1 and pax8, here referred to as the early nephrogenic transcription factors. However, the pathway inducing nephrogenesis and the network of theses factors are poorly understood. Treatment of the undifferentiated animal pole explant (animal cap) of Xenopus with activin A and retinoic acid induces pronephros formation providing a powerful tool to analyze key molecular events in nephrogenesis.

**Results:**

We have investigated the expression kinetics of the early nephrogenic transcription factors in activin A and retinoic acid treated animal caps and their potential to induce pronephric differentiation. In treated animal caps, expression of osr1, osr2, hnf1b and lhx1 are induced early, whereas pax8 expression occurs later implying an indirect activation. Activin A alone is able to induce osr2 and lhx1 after three hours treatment in animal caps while retinoic acid fails to induce any of these nephrogenic transcription factors. The early expression of the five transcription factors and their interference with pronephros development when overexpressed in embryos suggest that these factors potentially induce nephrogenesis upon expression in animal caps. But no pronephros development is achieved by either overexpression of OSR1, by HNF1B injection with activin A treatment, or the combined application of LHX1 and PAX8, although they influenced the expression of several early nephrogenic transcription factors in some cases. In an additional approach we could show that HNF1B induces several genes important in nephrogenesis and regulates lhx1 expression by an HNF1 binding site in the lhx1 promoter.

**Conclusions:**

The early nephrogenic transcription factors play an important role in nephrogenesis, but have no pronephros induction potential upon overexpression in animal caps. They activate transcriptional cascades that partially reflect the gene activation initiated by activin A and retinoic acid. Significantly, HNF1B activates the lhx1 promoter directly, thus extending the known activin A regulation of the lhx1 gene via an activin A responsive element.

## Background

During vertebrate development three kidney types of increasing complexity (pronephros, mesonephros and metanephros) form successively from the intermediate mesoderm, located between the paraxial mesoderm (developing somites) and the lateral plate [1]. The pronephros is the simplest, functional form of kidney in larval stages of fish and amphibians and consists of three major components: glomus, tubules and duct. In adults the pronephros is replaced by the mesonephros. In mammals the pronephros is not functional, but required for mesonephros formation that is replaced by the metanephros, the kidney of the adult [2].

All components of the pronephros arise from intermediate mesoderm, but the signals that direct pattering of the presumptive pronephric mesoderm towards pronephric lineages are unknown. Experiments showed that the anterior somites are crucial for pronephros development and provide an essential first signal. If the anterior somites are removed [3] or separated from the presumptive pronephros [4], pronephroi do not form. Anterior somites can also induce pronephric tubules in unspecified intermediate mesoderm [3]. Although the exact timing and nature of the signal provided by the anterior somites are yet unknown, wnt11b expressed throughout the anterior somites has recently been shown as a crucial signal [5].

Xenopus is a very attractive model organism to analyse key molecular events in nephrogenesis, because most genes essential for pronephros development in Xenopus embryos are also crucial for the formation of the more complex mammalian kidneys [6-9]. A classical method to identify important molecules in Xenopus development is the injection of mRNAs or morpholino oligonucleotides into the fertilized egg or into blastomeres of early cleaving stages [10,11]. Thus, several pronephric regulators have been functionally identified [7,8,12]. An additional experimental tool to study early events of nephrogenesis involves explanting the animal pole of the blastula. These explanted animal caps have pluripotency and differentiate into various tissues upon exposure to inducing substances [13,14]. Importantly, animal caps treated with activin A and retinoic acid differentiate into pronephros [15] and in this *in vitro *system genes are induced with similar kinetics as *in vivo *[16-18].

In Xenopus the first genes expressed in the pronephros anlage are the transcription factors osr1 and osr2, members of the odd-skipped family of proteins [19], hnf1b, a member of the homeobox factors [20], lhx1 (lim1), a lim homeobox factor [21] as well as pax8, a member of the paired box domain family [22]. We refer to these five transcription factors as the early nephrogenic transcription factors, as they are all expressed in the pronephros anlage prior to cellular differentiation and their misexpression affects pronephros development. Inhibition of osr1 or osr2 by morpholinos in Xenopus embryos interferes with kidney formation and embryonic overexpression of either of these factors induces ectopic kidney tissue and enlarged pronephros [19]. Overexpression of hnf1b inhibits pronephros formation [23] and this effect is also seen by using the human HNF1B [24] implying that the regulatory potential has been conserved during vertebrate evolution. In contrast, lhx1 and pax8 overexpression leads to an enlargement of the pronephros and, if both factors are coexpressed, this effect is increased and even induces ectopic pronephric tubules [25].

It should be noted that each of these five early nephrogenic transcription factors plays also a crucial role in the development of other organs. The prominent role of these nephrogenic transcription factors is partially also evident in mammalian systems. Whereas null mutation of Osr1 in mice exhibit agenesis of the kidney [26], Osr2 knock-out has no kidney phenotype [27], although Osr2 transcripts are expressed in the developing kidney [28]. The kidney-restricted knockout of Hnf1b leads to polycystic kidney disease [29] and the Lhx1 null mutant even lacks any kidney [30]. In contrast, Pax8 deficient mice exhibit thyroid gland deficiency, but have no pronephric phenotype [31]. Nevertheless, Pax8 plays an essential role in kidney development, as impaired metanephros formation observed in mice deficient for Pax2 [32] is dramatically increased by a lack of any nephric cell lineage, if these embryos lack additionally Pax8 [33].

To further explore the role of these five nephrogenic transcription factors we have now analyzed the kinetics of their induction in animals caps differentiated into pronephric tissue by activin A and retinoic acid. We then have overexpressed these transcription factors in animal caps and analyzed their potential to induce each other and to stimulate pronephric differentiation in these explants. To allow discrimination between injected mRNAs and endogenous mRNAs we used the human mRNAs that are functionally equivalent, but are not detectable with the Xenopus probes. We use capital letters for these human transcription factors to make a clear distinction. In addition we identified genes induced by HNF1B in these early embryonic cells.

## Results

### Induction of mRNAs encoding the early nephrogenic transcription factors osr1, osr2, hnf1b, lhx1 and pax8 in animal caps treated with retinoic acid and/or activin A

**Figure 1:**
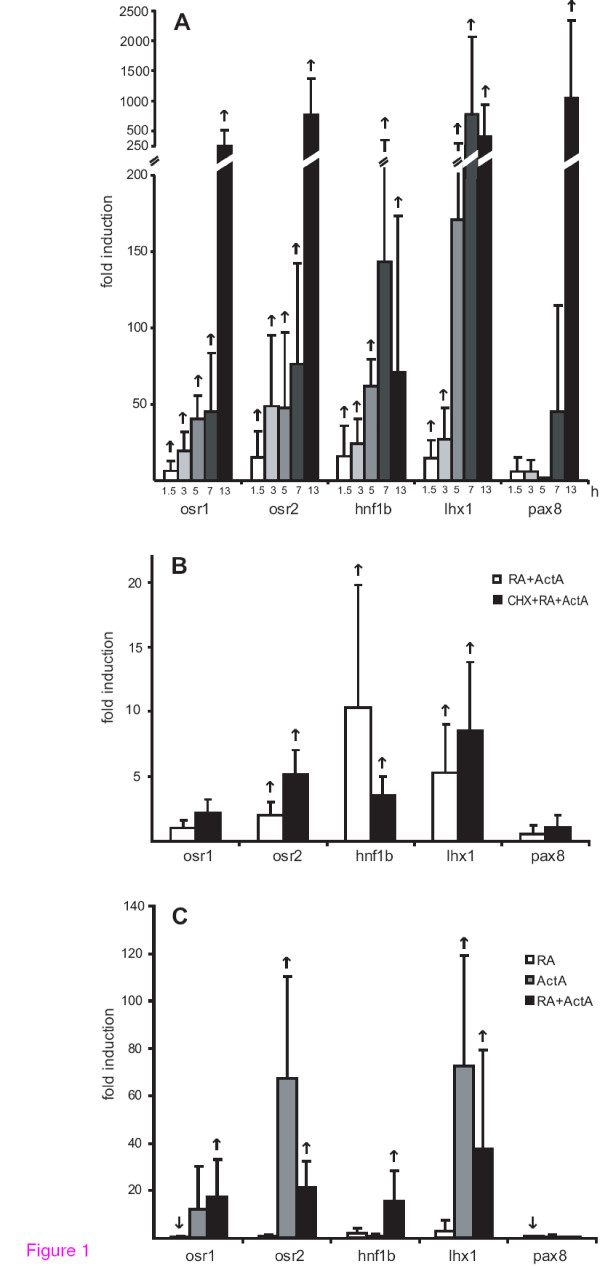
**Time dependent induction of the early nephrogenic transcription factors in retinoic acid and activin A treated animal caps**. **(A) **Animal caps were cut at the late blastula stage 9 [36] and incubated for 1.5 or 3 hours in retinoic acid (10^-4^M) and activin A (10 ng/ml). For 5, 7 and 13 hours treatments retinoic acid and activin A was replaced after three hours with Steinberg's solution [15]. The mRNA levels were analysed by quantitative RT-PCR using the primers given in Additional file 2. The standard deviations from four independent animal cap pools (N = 30) are given and alterations are indicated by an upward arrow, if all four induction values were higher than one for a given probe. To detect osr2 transcripts primers targeting osr2B, the splice variant predominantly expressed in Xenopus laevis (compare Figure 2Bs) were used for two animal cap pools analyzed after 3, 5 and 7 hours and three after 13 hours In all other experiments primers targeting both splice variants were used. **(B) **Animal caps cut at the late blastula stage 9 were incubated with or without cycloheximide (CHX, 5 μg/ml) for 30 min and then stimulated for 1.5 hours in retinoic acid (10^-4^M) and activin A (10 ng/ml). RNA was quantified from six pools (N = 30) and upward arrows indicate induction for all six experiments. **(C) **Animal caps were cultured for three hours in retinoic acid (RA, 10^-4^M), activin A (ActA, 10 ng/ml) or both inducers together (RA+ActA) and then analysed by quantitative RT-PCR. The standard deviations from five (RA) or four (ActA or RA+ActA) independent animal cap pools (N = 30) are given and reproducible induction or reduction is indicated by an upward or downward arrow, if increased or decreased in all samples, respectively.

Since simultaneous treatment of Xenopus animal caps with 10 ng/ml of activin A and 10^-4 ^M retinoic acid for three hours induces differentiation of pronephric tissues [15], we explored the time dependent induction of the early nephrogenic transcription factors osr1, osr2, hnf1b, lhx1 and pax8 in these embryonic explants. Thus, we measured by quantitative RT-PCR the induction of the corresponding mRNAs in animal caps treated with retinoic acid and activin A (Figure 1A). Based on the low sample pools (N = 4) and because we do not know whether the values obtained in various experiments with animal caps represent a normal distribution, the use of a significance test is not appropriate. Therefore, we defined genes as induced or repressed, when all the four measured values for a given sample pool represented the same trend. Using these criteria all five transcripts were induced, albeit with different kinetics. Whereas osr1, osr2, hnf1b and lhx1 mRNAs were induced within 1.5 hours, pax8 mRNA was only increased after thirteen hours. For osr1 and osr2 the induction increased up to thirteen hour treatment, whereas hnf1b and lhx1 seemed to level off at seven hour treatment. By inhibition of protein synthesis with cycloheximide, the induction of osr2, hnf1b and lhx1 was not abolished implying direct gene activation (Figure 1B). The observation that osr1 was not induced in this experiment possibly reflects the 30 min culture with or without cycloheximide prior to retinoic acid and activin A treatment, since delayed activin A treatment of animal caps reduces the induction potential [34].

To clarify which nephrogenic transcription factor transcripts are induced by retinoic acid or activin A alone, we analyzed animal caps treated with either retinoic acid or activin A for three hours (Figure 1C). Treatment with retinoic acid failed to induce the nephrogenic transcription factors, but rather decreased the level of osr1 and pax8 transcripts by 2-fold and 1.6-fold, respectively. In contrast, activin A treatment induced osr2 as well as lhx1, but osr1 in two of four experiments only. Since hnf1b was not induced by activin A alone, we conclude that a synergistic effect of both inducers is needed to induce hnf1b. The lack of induction of pax8 in animal caps treated with activin A or retinoic acid alone is consistent with the no-induction observed in animal caps treated with retinoic acid and activin A for three hours (also compare Figure 1A).

### Overexpression of OSR1 and Osr2A leads to enlargement of pronephros and ectopic pronephric tissue

**Figure 2:**
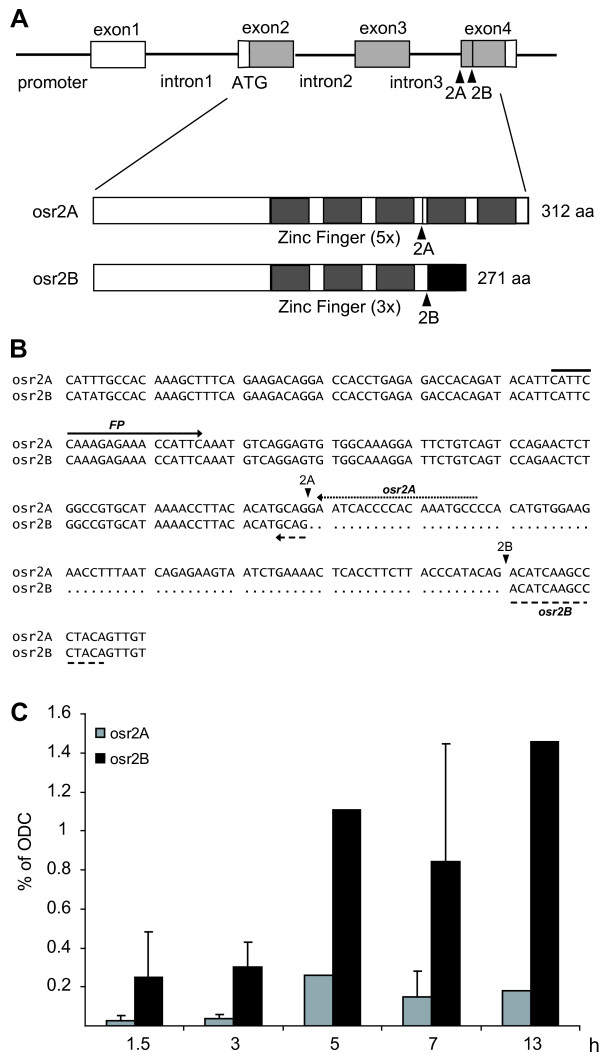
**Splice variants of Xenopus osr2**. **(A) **Schematic representation of the Xenopus osr2 splicing variants A and B based on the mammalian data [35] shows alternative splice sites 2A and 2B indicated by arrowheads. Boxes represent exons and filled areas reflect open reading frames, while lines represent the promoter and introns. In the lower panel a scheme of the five and three zinc-finger domains (grey boxes) in osr2A and in osr2B protein is given, respectively. The black box in osr2B denotes the alternatively spliced exon 4. **(B) **Alignment of the nucleotide sequence around alternatively spliced exon 4 is based on a Xenopus laevis osr2B full-length cDNA sequence (accession no. BC108579), whereas the corresponding osr2A cDNA of Xenopus tropicalis (accession no. CU075721) is taken, as no corresponding Xenopus laevis cDNA is available. The nucleotide sequence around alternatively spliced sites 2A and 2B (indicated by arrowheads) is conserved in both species. The forward primer FP is identical for both splice variants, whereas the reverse primers distinguish between osr2A and osr2B. **(C) **Animal caps were cut at the late blastula stage 9 [36] and incubated for 1.5 or 3 hours in retinoic acid (10^-4^M) and activin A (10 ng/ml). For 5, 7 and 13 hours treatments retinoic acid and activin A was replaced after three hours with Steinberg's solution as originally described [15]. The mRNA levels were analysed by quantitative RT-PCR using the specific primers for osr2A and osr2B (see Additional file 2) and the results are normalized to odc expression levels. If two independent pools were analysed, the mean is given and the bar marks the two values.

Since the murine Osr2 gene is expressed in two splice variants referred to as Osr2A and Osr2B [35], we searched Xenopus cDNA sequences deposited in GeneBank for corresponding splice variants. Indeed, we identified transcripts encoding the A and B splice variants that encode a five and three finger zinc protein, respectively, comparable to the murine situation (Figure 2A and 2B). Using primers specific for the A and B splice variant we could show that osr2B predominates osr2A by a factor of about three throughout retinoic acid and activin A induction in animal caps (Figure 2C).

**Figure 3:**
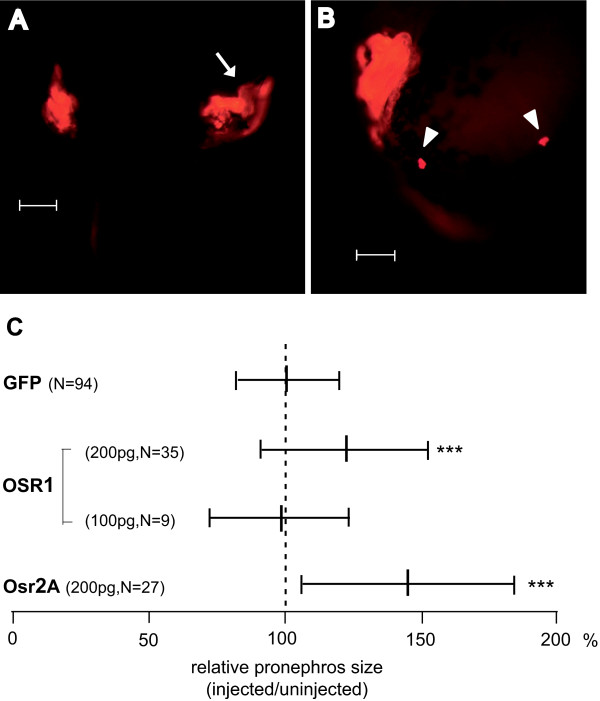
**Expression of OSR1 and Osr2A leads to an increase of pronephros size in Xenopus laevis larvae**. **(A) **Enlargement of pronephros: Dorsal view of stage 45 larvae [36] injected with 200 pg OSR1 mRNA and whole-mount immunostained with antibodies 3G8 and 4A6 [37]. The injected side is marked by an arrow. Bar = 200 μm **(B) **Ectopic kidney cell patches (marked by arrow heads): Lateral view of a stage 45 larvae [36] expressing OSR1 and prepared as in A. Bar = 200 μm **(C) **Pronephros size on injected versus control side in OSR1 or Osr2A injected embryos was determined in lateral views by measuring the area through the widest part of the immunostained pronephros. The Student's t-test was used to score significant differences. The p-value is given by comparing embryos injected with gene of interest plus GFP mRNA as marker with those injected with GFP mRNA taken from previous data [23]. _*** _denotes p-value ≤ 0.001. The vertical line in the middle represents the mean and the small bars the standard deviation. N is the number of animals investigated.

To examine the morphogenetic potential of the mammalian Osr1 and Osr2A in developing Xenopus embryos, we injected mRNA encoding the human OSR1 or the murine Osr2A proteins into one blastomere of the two-cell stage embryo using GFP mRNA as a marker to identify the injected side. About half of the OSR1 and more than the half of the Osr2A injected embryos showed gastrulation defects that were more severe with higher doses of mRNA. Injected embryos surviving to the free swimming tadpole stage 45 [36] were immunostained for pronephros development using a mixture of the monoclonal antibodies for the proximal tubules (3G8) and the distal tubules and pronephric duct (4A6) [37]. The majority of embryos injected with OSR1 showed an enlarged pronephros (Figure 3A), in some cases together with the formation of ectopic kidney cell patches (Figure 3B). Comparing the size of the pronephros of the injected side with the one of the non-injected side we observed that the pronephros size was significantly enlarged by injecting 200 pg OSR1 but not with 100 pg (Figure 3C). Similarly, Osr2A overexpression (200 pg) leads to a significant increase of pronephros size as well, but we did not observe the formation of ectopic kidney tissue. In conclusion, OSR1 as well as Osr2A overexpression induces pronephros enlargement in Xenopus embryos as found previously for the corresponding Xenopus factors osr1 and osr2B suggesting comparable function of both mammalian genes in pronephros development [19].

### Overexpression of OSR1 alone or in combination with retinoic acid or activin A cannot support pronephros differentiation in animal caps

**Figure 4:**
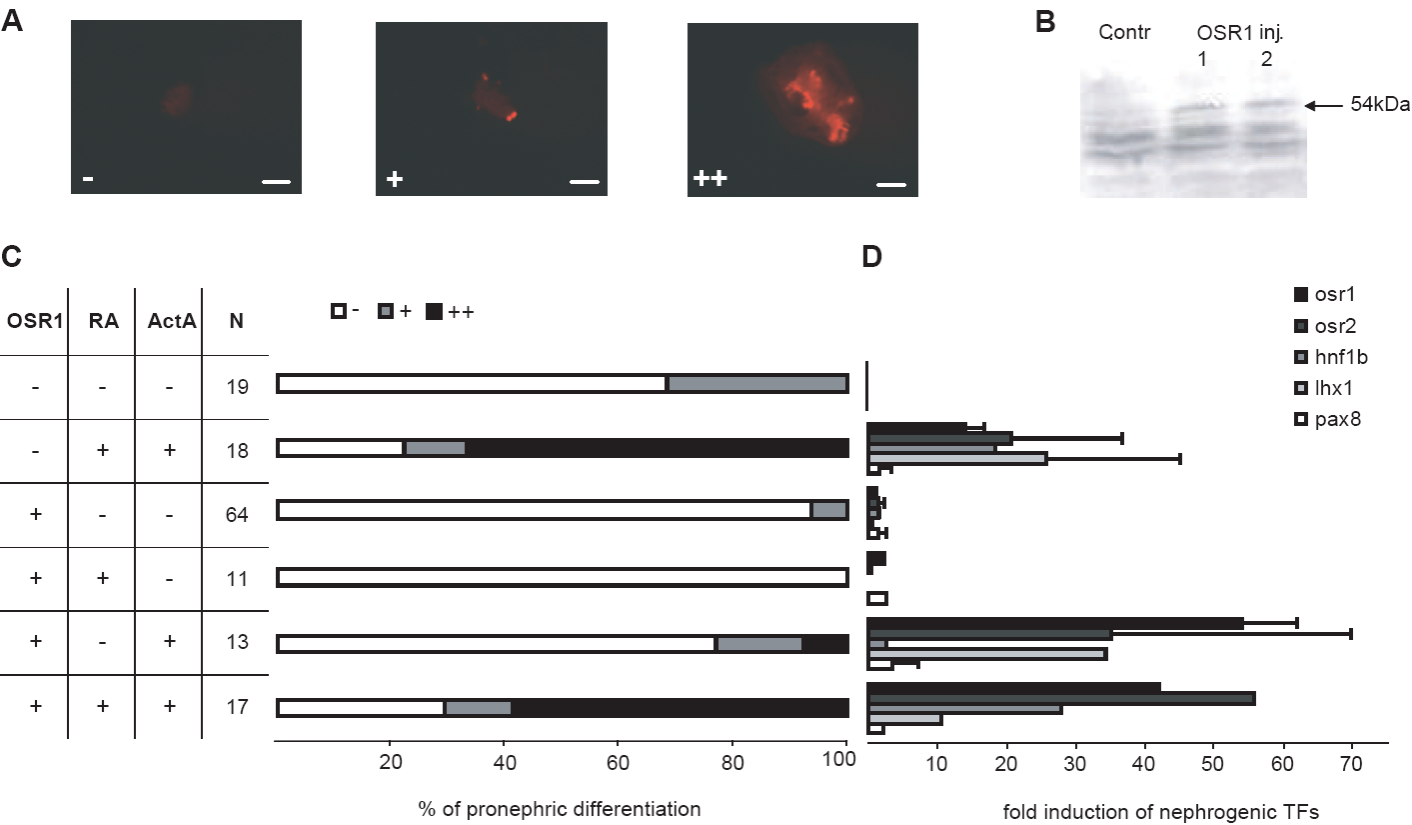
**Expression of human OSR1 in animal caps**. Animal caps derived from embryos injected with OSR1 mRNA were cultured in Steinberg's solution, retinoic acid (RA, 10^-4^M) and/or activin A (ActA, 10 ng/ml) as indicated. To avoid a too high lethality only 150 pg mRNA was injected. **(A) **Classification of whole-mount immunostained animal caps used for the analysis of pronephric tissue induction. The immunostaining was done with a mixture of the antibodies 3G8 and 4A6: - no pronephric tissue, + two or more pronephric cell patches or a small area of pronephric cells, +++ pronephric tubule like structures. Bar = 200 μm **(B) **Exogenous OSR1 is expressed in injected embryos. After cutting the animal caps protein extracts of the remaining late blastula stage embryos injected with OSR1 mRNA were used for Western blot analysis with myc-tag specific antibody. Lane 1 and 2 refer to two pools of ten embryos. The exogenous myc-OSR1 is marked by an arrow. **(C) **The pronephros induction potential in animal caps was assayed after four days using the categories given in panel A. N refers to the number of animal caps investigated. **(D) **Quantitative RT-PCR analysis for the expression of early nephrogenic transcription factors (TFs) of animal caps after three hours and treated as indicated in (C). The bar represents the values of two independent animal cap pools (N = 30) tested. The results of OSR1 injection with retinoic acid treatment alone or in combination with activin A are each from one animal cap pool. Data obtained for hnf1b expression were only from one pool tested except for OSR1 injection alone.

Since in our experiments expression of OSR1 had a lower lethality compared to Osr2A and induces ectopic kidney tissues (Figure 3B), we investigated the potential of OSR1 to trigger pronephric differentiation in animal caps. Therefore, we injected mRNA encoding human OSR1 into the animal pole of two-cell stage embryos, explanted animal caps at late blastulae and cultured the explants for four days to monitor pronephros differentiation by immunostaining with the pronephros specific antibodies. In a control experiment without mRNA injection, but adding retinoic acid as well as activin A, pronephric differentiation was observed. Since the extent of differentiation was quite variable between individual caps, we scored the explants into three categories as illustrated in Figure 4A. Using the distribution into these categories it is clear that retinoic acid combined with activin A induced pronephric differentiation compared to untreated animal caps (Figure 4C), demonstrating kidney differentiation *in vitro *as previously reported [15]. However, OSR1 overexpressing animal caps failed to differentiate and this was not improved by adding retinoic acid or activin A. Furthermore, OSR1 overexpression in animal caps treated with retinoic acid and activin A led to a similar extent of pronephric differentiation compared to uninjected, but treated animal caps. Quantitative RT-PCR analysis after three hours showed no induction of the early transcription factors upon OSR1 expression alone or in combination with retinoic acid, whereas treatment of OSR1 injected caps with activin A led to an induction of osr1, osr2 and lhx1 (Figure 4D), as observed frequently by activin A treatment alone (Figure 1C). As a control we verified the presence of the OSR1 protein translated from the injected OSR1 mRNA by Western blots (Figure 4B). Taken together, OSR1 alone or in combination with retinoic acid or activin A does not have the potential to induce pronephros differentiation in animal caps.

### LHX1 and/or PAX8 or HNF1B are not sufficient to induce pronephros differentiation in animal caps

**Figure 5:**
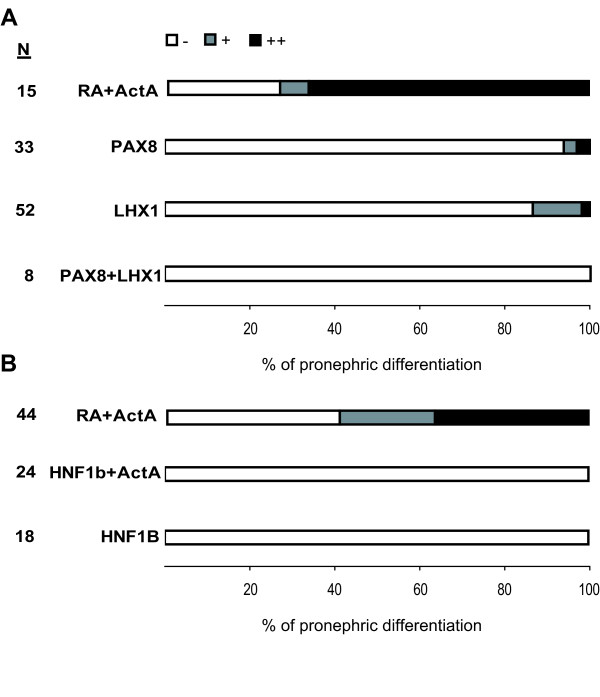
**Expression of human LHX1, PAX8 and HNF1B in animal caps**. Animal caps of LHX1 and/or PAX8 (250 pg alone or 125 pg each) or HNF1B (150 pg) injected embryos were cultured in Steinberg's solution for three hours and analysed after four days by whole-mount immunostaining using a mixture of the antibodies 3G8 and 4A6. Activin A (ActA, 10 ng/ml) and retinoic acid (RA, 10^-4^M) were added as given. **(A) **and **(B) **The pronephric tissue induction in animal caps treated as indicated on the left was scored using the categories given in Figure 4A. The number of animal caps (N) is given.

It is known that embryonic overexpression of Xenopus lhx1 or pax8 induces enlargement of pronephros and coexpression of both factors leads to a synergistic effect [25]. This enlargement of the pronephros we also observed by unilateral injection of the human transcription factors, since coinjected LHX1 and PAX8 result in a pronephros size of 163 +/- 42% compared to the control side (N = 23, p-value = 3 × 10^-7^). However, in contrast to experiments using the Xenopus factors [23,25], no ectopic pronephric tissue formation was seen with the human factors. To investigate the pronephros differentiation potential of human LHX1 and PAX8 in animal caps, we injected mRNA encoding these proteins, either alone or in combination, into the animal pole of two-cell stage embryos and analysed the animal caps by immunostaining after four days. Both LHX1 and PAX8 alone or in combination could not induce pronephros differentiation in animal caps at day four (Figure 5A). Measuring the level of the transcripts encoding the five nephrogenic transcription factors after 3 hours we observed no change (data not shown). The missing pronephros formation in animal caps overexpressing LHX1 and PAX8 is consistent with unpublished data reviewed recently [14]. To prove successful injection, we confirmed in all embryos green fluorescence derived from the coinjected GFP mRNA. In addition, for LHX1 injections into animal caps we deduce functional relevant amounts of LHX1 protein, as the lhx1 target gene cerberus (cer1) [38] was 6- or 64-fold induced in two independent experiments (data not shown).

Since differential addition of retinoic acid and activin A to animal caps has shown that activin A alone induces osr2, lhx1 and frequently also osr1, but never hnf1b that requires retinoic acid in addition (Figure 1C), we wondered whether HNF1B injection might replace retinoic acid to get pronephric induction in animal caps. However, injection of HNF1B mRNA into the animal pole at the two-cell stage, failed to induce pronephric differentiation in the explanted animal caps, even if cultured in the presence of activin A (Figure 5B). In conclusion, LHX1, PAX8 and HNF1B failed to induce pronephros differentiation in animal caps.

### Overexpression of HNF1B induces in animal caps genes important for nephrogenesis

Since numerous target genes of hnf1b have been defined [39] or postulated [40] in various mammalian systems, we wonder whether these genes are activated in animal caps representing embryonic stem cells. Thus, we searched for orthologs present in Xenopus ESTs and selected 26 genes reported to be expressed early in Xenopus development or in the developing pronephros [41]. Analysing animal caps of HNF1B injected embryos by quantitative RT-PCR revealed after seven hours a clear increase in transcripts encoding the early nephrogenic transcription factors lhx1, osr2 and osr1, but also of those encoding the transcription factors hnf1a, hnf4a and tfe3 as well as the signalling molecules wnt11b and gdnf (Table 1). After fourteen hours we found as a delayed response induction of the transcripts encoding the transcription factor pax2 and esterase D (esd). At this later time point decreased level of transcripts encoding the RNA binding protein rbms1 and the fibroblast growth factor receptor (fgfr4c) were found implying secondary effects. As lhx1 transcripts are induced by hnf1b, we also tested five transcripts of genes known to be targeted by lhx1. Indeed, cerberus (cer1) and chordin (chrd) were induced, but three other lhx1 targets were not (Table 1). In fact, goosecoid (gsc) was downregulated after fourteen hours. Taken together, HNF1B can activate in animal caps several genes involved in kidney development, but some genes considered to be HNF1B targets in mammals are not influenced.

**Table 1:** Activation of genes by HNF1B in animal caps

	gene symbol	fold induction	
		7 h (N = 5)	14 h (N = 4)
early nephrogenic TFs	**lhx1**	**9.9 **↑	1.5
	
	**osr2**	**4.3 **↑	2.2
	
	**osr1**	**3.7 **↑	2.5
	
	**pax8**	2.4	1.7
	
	**hnf1b**	1.3	0.9

genes involved in nephrogenesis	**hnf1a**	**13.7 **↑	7.1
	
	**wnt11b**	**4.6 **↑	1.3
	
	**wnt11**	1.6	1.1
	
	**pax2**	1.1	**10.7 **↑
	
	**gdnf**	**1.2 **↑	1.4
	
	**wnt9b**	0.8	0.7

proximal tubule genes	**prodh2**	95.9	1.7
	
	**hnf4a**	**22.9 **↑	3.4
	
	**anxa13**	5.3	1.4
	
	**slc22a6**	3.4	25.6
	
	**cpn1**	2.3	3.1
	
	**tmem27**	2.2	3.5
	
	**slc5a2**	1.9	2.3
	
	**tfe3**	**1.8 **↑	1.2
	
	**rpl35a**	1.5	1.5
	
	**gjb1**	1.3	1.0
	
	**trps1**	1.2	0.8
	
	**ube3a**	1.1	0.8
	
	**esd**	1.0	**1.3 **↑
	
	**rbms1**	1.0	**0.2 **↓
	
	**slc7a8**	1.0	1.5
	
	**fgfr4a**	0.9	0.7
	
	**c8a**	0.9	1.1
	
	**ncor1**	0.9	1.1
	
	**slc4a7**	0.9	1.1
	
	**fgfr4c**	0.7	**0.5 **↓

lhx1 target genes	**cer1**	**421.3 **↑	2.9
	
	**chrd**	**9.0 **↑	7.8
	
	**pcdh8.2**	5.1	2.0
	
	**gsc**	3.5	**0.1 **↓
	
	**otx2**	1.3	1.3

HNF1B (150 pg) injected animal caps were cultured for seven or fourteen hours in Steinberg's solution and analysed by quantitative RT-PCR for gene expression of the early nephrogenic transcription factors, of genes involved in nephrogenesis [78], of proximal tubule genes [40] and of the lhx1 target genes chrd [82], cer1 [38], gsc [82], otx2 [83] and pcdh8.2 [84] using the primers given in Additional file 2. Based on the low sample numbers (N = 4 or 5), genes were defined as induced or repressed, if all induction values were either higher or lower for a given probe. Distinct induction or reduction is indicated by an upward or downward arrow, respectively. N is the number of pools with 30 animal caps.

### HNF1B regulates lhx1 transcription by an HNF1 binding site in the lhx1 promoter

**Figure 6:**
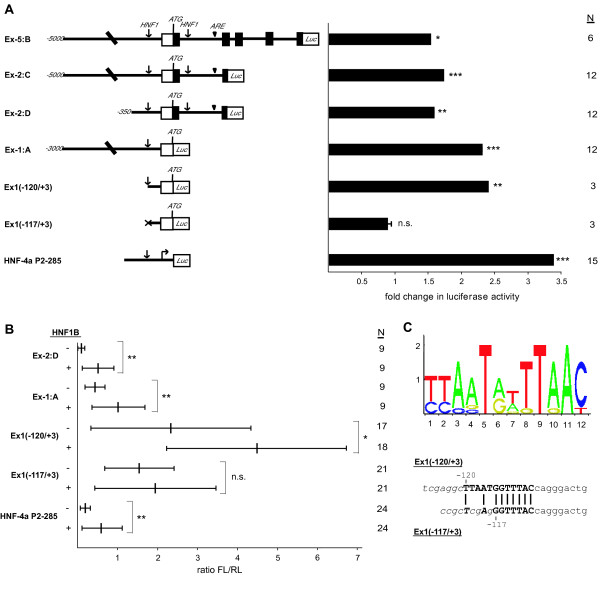
**Functional identification of HNF1 binding sites in the promoter region of lhx1 in HEK293 cells and in animal caps**. **(A) **HEK293(HNF1B) cells [45] were transfected with lhx1 gene firefly luciferase fusion constructs and HNF1B expression was induced by adding doxycycline. On the left panel schematic drawings of transfected lhx1 promoter luciferase fusion constructs (not to scale) are given. Lines represent promoter and intron regions, boxes are exons with protein coding (filled) or untranslated (open) regions (adapted from [42]). Arrows mark potential HNF1 binding sites identified by JASPAR [44]. The arrow head marks the ARE (activin response element) in intron 1 [42]. The Ex1(-120/+3) construct contains the complete HNF1 binding site in the promoter region of lhx1, whereas the binding site in the Ex1(-117/+3) construct is partially deleted (compare panel C). The HNF-4a P2-285 construct contains the P2-promoter of the HNF-4a gene which is regulated by HNF1B [46]. The fold change of luciferase activity of transfected constructs in doxycycline induced HEK293(HNF1B) versus untreated cells is given using the *renilla *luciferase reporter pRL-Con as an internal control. The Student's t-test was used and *, ** and *** refer to p-value of ≤ 0.05, ≤ 0.01 and ≤ 0.001, respectively and n.s. means not significant. N is the number of independent transfections made. **(B) **The luciferase reporter constructs (50 pg each) were tested in animal caps of controls or HNF1B (150 pg) injected embryos. The luciferase activity was measured in pools of four animal caps after four hours cultivation in Steinberg's solution. To calculate the increase of luciferase activity in HNF1B injected animal caps the ratio of *firefly *luciferase (FL) to *renilla *luciferase (RL) was used (FL/RL). The vertical line in the middle represents the mean and the small bars the standard deviation. Since experiments with different pools of animal caps were not comparable in quantitative terms, we used the Mann-Whitney-test to score significant differences. * and ** refer to p-value of ≤ 0.05 and ≤ 0.01, respectively and n.s. means not significant. N is the number of animal cap pools tested. **(C) **The upper panel shows the sequence logo of HNF1 binding site given in JASPAR [44] and the lower panel the sequences of the HNF1 binding site (capital letters) in Ex1(-120/+3) and Ex1(-117/+3) constructs. The vector sequence is indicated by italics.

The Xenopus lhx1 gene is known to be regulated in early embryogenesis by activin A via an activin response element (ARE) present in the first intron [42,43]. Since our data show that HNF1B induces lhx1 transcripts in animal caps without activin treatment, we explored a direct activation of the lhx1 gene via HNF1B. By *in silico *analysis with JASPAR [44] we identified potential HNF1 binding sites (Figure 6C) in the promoter region and the first intron of the Xenopus lhx1 gene (Figure 6A). To determine functional HNF1 binding sites, we tested four luciferase reporter constructs carrying various fragments of the lhx1 gene (Figure 6A). These constructs were transiently transfected into a HEK293 cell line containing tetracycline-inducible HNF1B [45]. As shown in Figure 6A, the construct Ex-5:B containing the entire lhx1 gene was inducible by HNF1B and by analysis of various deletion constructs regulation by HNF1B could be pinned down to the HNF1 site of the promoter area (compare Ex1(-120/+3) versus Ex1(-117/+3) in Figure 6A). Clearly, partial mutation (three of twelve base pairs) of the HNF1 site of the promoter (Figure 6C) abolished HNF1B regulation completely. HNF1B transactivation via the HNF1 binding site in the lhx1 promoter is about 70% (Figure 6A) compared to the effect seen with the HNF4A P2 promoter, a well studied HNF1B target [46,47].

To extend these findings to Xenopus embryonic cells we tested some constructs in animal caps that were derived from controls or HNF1B injected eggs. All constructs retaining the HNF1 binding site were transactivated by injected HNF1B, including the minimal construct Ex1(-120/+3), whereas the reporter Ex1(-117/+3) containing the partially deleted HNF1 site was not inducible (Figure 6B). From these results we conclude that the lhx1 promoter carries a functional HNF1 binding site that is active in HEK293 cells as well as in embryonic cells of Xenopus.

## Discussion

Animal caps are a suitable system to analyse nephrogenesis *in vitro*, because pronephros differentiation can be induced by treatment with activin A and retinoic acid [15]. Activin A simulates as a TGF-β family member the vegetalizing factor 1 (Vg1) [48]. This factor whose maternal mRNA is localized to the vegetal pole of Xenopus eggs [49,50] has mesoderm-inducting activity and is an essential regulator of embryonic patterning [51]. On the other hand retinoic acid regulates major embryonic growth and patterning decisions and its availability is regulated by synthesizing and metabolizing enzymes [52]. In Xenopus retinal dehydrogenase (RALDH2) creates a critical retinoic acid concentration gradient along the anteroposterior axis [53] and it was shown that retinoic acid treatment of embryos leads to larger pronephros [54], whereas defective retinoic acid signalling impairs pronephros development [55].

Although the animal cap assay represents a powerful system to analyse key molecular events in nephrogenesis, it has some limitations, since pronephros differentiation does not occur in all animal caps. In our hands treated animal caps revealed pronephric induction rate of about 60-85% comparable to about 80% described previously [15,18]. Significantly, animal caps often died during the four day incubation and this lethality was increased upon mRNA injection, but a clear activation of pronephros differentiation was seen in surviving activin A and retinoic acid treated animal caps. The inhomogeneous response of individual animal caps seen by antibody staining was also observed when comparing the induction of specific transcripts between different experiments (Figure 1 and 4). This experimental variation was also evident in transactivation of promoter luciferase reporter constructs (Figure 6B), a limitation reported previously [42,56]. In these experiments the variable outcome is possibly further increased, since reporter constructs and mRNAs cannot be introduced at exactly the same level into each animal cap. In spite of these technical difficulties, we successfully used the animal caps to identify several transcriptional regulatory pathways in this differentiating system.

Our analysis of animal caps treated with activin A and retinoic acid revealed the known induction of lhx1 (Taira et al., 1992) and pax8 [22], but also induced expression of osr1, osr2 and hnf1b (Figure 1A). Interestingly, in animal caps the kinetics of induction reflects the expression *in vivo*, an observation made for other induced RNAs previously [16,57]. Thus, the expression of osr1, osr2, lhx1 and hnf1b after 1.5 hours treatment corresponds with their embryonic expression at early gastrula [19-21] and pax8 expressed later in animal caps agrees with its expression in late gastrula [25].

In animal caps activin A can induce osr2 and lhx1 alone reflecting its strong inducing activity in animal caps [58]. In contrast retinoic acid fails to induce any of the five factors (Figure 1C) correlating the fact that retinoic acid does not induce pronephros [13,59,60]. Previous experiments showed a low lhx1 induction in retinoic acid treated animal caps [21,61] that possibly reflect subtle differences in the induction protocol or the animals used.

The fast induction of osr2, hnf1b and lhx1 reflects most likely a direct activation by activin A and retinoic acid, since the induction of these transcripts was not inhibited by cycloheximide treatment (Figure 1B), a finding that has been previously reported for activin A induced lhx1 transcripts [21,62]. Consistent with such a direct activation an actvin A respose element (ARE) has been identified in the lhx1 promoter [42,43].

Previous experiments showed that it is possible to induce tissue differentiation by overexpressing transcription factors in animal caps. For instance overexpressed Xbra leads to mesoderm differentiation with muscle, mesothelium and mesenchyme [63,64], whereas Sox1 [65] or Zic3 [66] induce neural tissues. In contrast, overexpression of the five nephrogenic transcription factors failed to trigger pronephros differentiation in animal caps (Figure 4 and 5). However, as we used only the common pronephric markers 4A6 and 3G8, we cannot exclude that some other pronephric differentiation products are induced.

In other experiments the failure of injected transcription factor to induce differentiation in animal caps could be overcome by adding growth factors. For example the induction of neural crest differentiation by Pax3 and Zic1 requires Wnt signaling [67] or Neptune induces erythropoiesis only together with GATA1 and bFGF [68]. Concerning pronephros differentiation the ectodermal character of the animal pole cells has possibly first to be changed to a mesodermal fate by adding mesoderm inducing factors. Hence, we added activin A to OSR1 treated animal caps, but without enhancing pronephros differentiation of animal caps (Figure 4C). Since Osr1 has essential functions at the beginning of kidney development [26,69] and the ability to induce the three early nephrogenic transcription factors hnf1b, lhx1 and pax8 *in vivo *[19], its inability to induce pronephros differentiation is striking. However, murine cells expressing Osr1 although multipotent and necessary to build the metanephric precursors require signals from the surrounding tissues for kidney development [70]. Similarly in zebrafish osr1 is required to limit endoderm differentiation to allow kidney development [71]. Since OSR1 did not improve the differentiation potential of activin A and retinoic acid treated animal caps (Figure 4C), other signalling molecules are missing. In this context it may be relevant that ectopic kidney tissues in OSR1 (Figure 3B), osr2 (Tena et al., 2007) or lhx1 and pax8 [23,25] overexpressing embryos were found exclusively close to the pronephros. This suggests that signals in the region of pronephros anlage are needed. Most recently Wnt11b has been proposed as such a signal [5].

In our experiments we overexpressed either the human or murine transcription factors in order to distinguish the activity of the endogenous gene from the injected RNA, because previous experiments have shown equivalence between Xenopus and human hnf1b. This we confirmed in principle for OSR1 and Osr2A (Figure 3) as well as for coinjected LHX1 and PAX8 (see text). However, in contrast to the Xenopus factors, human LHX1 coinjecetd with PAX8 cannot induce ectopic pronephric tissue and such subtle differences may limit the use of mammalian factors in Xenopus embryos.

Investigating the influence of HNF1B on 26 potential hnf1b target genes we could show the activation of nine genes in injected animal caps (Table 1). The induced genes include the transcription factor lhx1, hnf1a, hnf4a and tfe3 suggesting the activation of various transcriptional cascades. This assumption is supported by the observation that the lhx1 target genes cer1 [38] and chrd [72], both with important roles in nephrogenesis, were also induced. Since other lhx1 target genes were not activated (Table 1), we assume that some lhx1 target genes are inhibited by HNF1B expression. It is striking that cer1 is induced at very high levels and peaked off within seven hours indicating a transient activation of cer1 by lhx1 which itself is only transiently induced by HNF1B (Table 1). The activation of hnf1a was expected, as HNF1B expression in the Xenopus embryos activates hnf1a [24] and the hnf1a promoter contains a functional HNF1 site [73]. Since HNF1B induces the expression of osr1 and osr2 we deduce a positive feedback loop, as osr1 and osr2 are able to induce hnf1b [19]. Furthermore, an increased expression of the signalling molecules wnt11b [5] and gdnf [74] were found, both of which play a role in nephrogenesis. Similarly, the downregulation of the fibroblast growth factor receptor (fgfr4c) may be relevant, as fgfr4c down-regulation is needed to allow pronephros development [75]. The delayed induction of two other important molecules in nephrogenesis, pax2 [76] and esd [77], implied an indirect activation. The fact that several genes crucial for pronephros development and expressed later in more differentiated pronephric tissues were not activated in animal caps by HNF1B, may either reflect that hnf1b acts differentially in Xenopus compared to mammals or more likely other signals are missing in the undifferentiated animal cap. Albeit our data show that HNF1B regulates in embryonic cells many genes potentially essential for pronephros development.

A function of Hnf1b in early nephrogenesis has most recently been reported also in mice by tetraploid and diploid embryo complementation to overcome early embryonic lethality of homozygous knock-out mice [78]. Significantly, in these mice Lhx1 was shown to be regulated by Hnf1b and to contain an HNF1 binding site in the far upstream promoter. Thus, the regulation of lhx1 by hnf1b seems to be evolutionary conserved, as a 10-fold increase in the expression level of lhx1 in HNF1B differentiated animal caps is seen (Table 1) and this induction is mediated by an HNF1 binding site in the Xenopus lhx1 promoter region (Figure 6).

## Conclusions

**Figure 7:**
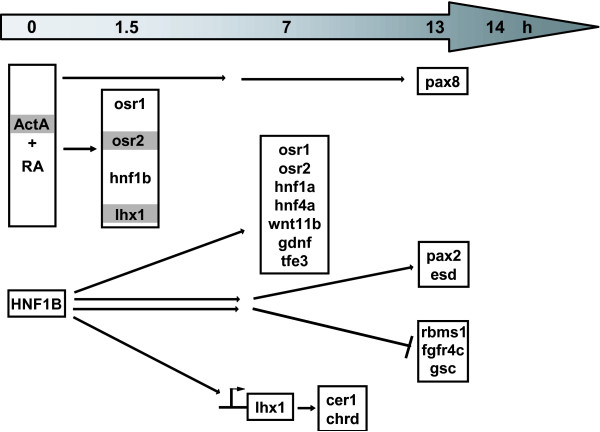
**Induction of the early nephrogenic transcription factors and other genes in animal caps**. osr1, osr2, hnf1b and lhx1 are induced already after 1.5 hours treatment with activin A (ActA) and retinoic acid (RA) suggesting that they are direct targets, whereas pax8 is first expressed after thirteen hours implying an indirect activation. osr2 and lhx1 (highlighted in grey) are induced after three hours by activin A alone. HNF1B overexpressed in animal caps induces after seven hours osr1, osr2, hnf1a, hnf4a, wnt11b, gdnf and tfe3. After fourteen hours pax2 and esd are increased and rbms1, fgfr4c and gsc are decreased suggesting secondary effects. HNF1B induces the expression of lhx1 by an HNF1 binding site in its promoter region (arrow). Furthermore, the lhx1 target genes cer1 and chrd are induced in HNF1B injected animal caps possibly via lhx1.

In this study using animal cap assays we provide insight into the network of the early nephrogenic transcription factors in pronephros development and the genes induced by HNF1B, especially (Figure 7). Significantly, we identified a functional HNF1 binding site in the lhx1 promoter. However, none of the early nephrogenic transcription factors has the ability to induce pronephros differentiation in animal caps.

## Methods

### Plasmids

The myc-Rc/CMVHNF1B expression vector has been described previously [24]. The mouse Osr2A plasmid was kindly provided by S. Kawai (Osaka University Graduate School of Dentistry, Osaka, Japan). The full-length open reading frame of the human gene OSR1 (IRATp970D0444D6; RZPD, Gemany) was cloned into pCS2+MT [79] to generate N-terminal myc-tag fusion proteins using 5'-GCTCTAGAGATGGGCAGCAAAACCTTGCC-3' and 5'-GCTCTAGATTAGCATTTGATCTTGGAGGTTTT-3' as forward and reverse primers, respectively. The XbaI restriction sites for cloning are underlined and the construct was verified by sequencing. The full-length open reading frames of the human PAX8 (RC200651) and LHX1 (RC210977) in pCMV6 were obtained from OriGene Technologies.

The pRL-Con renilla luciferase construct [80] and the HNF-4a P2-285 pGL3-Basic reporter plasmid [46] have been described. The lhx1 gene luciferase fusion constructs Ex-1:A, Ex-2:C, Ex-2:D and Ex-5:B [42] were kindly provided by M.L. Rebbert (NICHD, USA). The lhx1 reporter construct harbouring the HNF1 binding site in the promoter region (Ex-2:C) was used for additional constructs. XhoI/HindIII DNA fragments containing the intact or mutated HNF1 binding site in the promoter region to the transcription start generated by PCR using the primers Ex1(-120/+3): 5'-CCGCTCGAGGCTTAATGGTT-3' (forward), Ex1(-117/+3): 5'-CCGCTCGAGGGTTTACCAG-3' (forward) and 5'-CCCAAGCTTTCCCTTTGGTTAT-3' (reverse) were inserted into the XhoI and HindIII digested pGL3-Basic vector (Promega). The restriction sites for cloning are underlined. Both constructs were verified by sequencing.

### mRNA injection

The expression vectors encoding OSR1, Osr2A, HNF1B, LHX1 and PAX8 proteins and the GFP encoding expression vector (pCSGFP2) were linearized and *in vitro *transcribed with RNA polymerases (Nielsen and Shaprio, 1986). The restriction enzymes and RNA polymerases used are given in Additional file 1. Capped mRNA encoding the different proteins together with 100 pg of capped green fluorescent protein (GFP) mRNA as internal control were injected into one blastomere of the two-cell stage and after two days, the injected side was scored under a stereofluorescence microscope for the presence of GFP. In case of animal explants, the capped mRNA together with GFP is injected into the animal region of Xenopus embryos in each blastomere of the two-cell stage.

### Animal cap assays

Xenopus late blastulae, stage 9 [36], were de-jellied by treatment with 2% cysteine hydrochloride in water. The presumptive ectoderm (animal cap) was isolated with loops of 20 μm platinum wire heated to about 450°C for a few microseconds using the Gastromaster (Xenotek Engineering, Belleville, USA). The explants were incubated for three hours in Steinberg's solution (58 mM NaCl, 0.67 mM KCl, 0.34 mM Ca(NO_3_)_2_, 0.83 mM MgSO_4_, 3 mM HEPES, pH 7.8) containing recombinant human activin A (10 ng/ml; Sigma, A4941) and all-trans retinoic acid (10^-4^M; Sigma, R2625) or only in Steinberg's solution for controls and for explants from mRNA injected embryos. After three-times washing with Steinberg's solution the explants were cultured in Steinberg's solution at 20°C until they were equivalent to stage 40-42 in normal embryos (four days) and used for whole-mount immunostaining.

### Quantitative RT-PCR

RNA from pools of 30 animal caps was isolated with peqGold RNAPure (PeqLab) followed by phenol/chloroform extraction. For cDNA synthesis the High Capacity cDNA Reverse Transcription Kit (Applied Biosystems) was used. SYBR-Green real-time PCR was performed on a 7900HT Sequence Detection System (Applied Biosystems) using Power-SYBRGreen Mix (Applied Biosystems). Templates were determined in duplicate and for primers used see Additional file 2. Results are normalized to ornithin decarboxylase (odc) expression levels. In all cases water only and reverse transcriptase negative controls failed to produce specific products. The fold induction of the early transcription factors was obtained by comparison of treated and untreated animal caps.

### Whole-mount immunostaining

Whole-mount immunostaining of four day cultured animal caps or animals at the swimming larval stage were done as described [81]. The difference between the injected and the non-injected sides of embryos was evaluated by measuring the whole area using the lateral view with the widest diameter from the dorsal to the ventral side of the immunostained pronephros including the pronephric tubules and the anterior part of the pronephric duct. The measurements were made using AxioVision 4.6 software (Carl Zeiss Imaging Solutions), and the non-injected side was used as a reference for each animal. The values representing kidney size obtained from each mRNA injected embryo were compared to values obtained from GFP control-injected embryos (data adapted from [23]). Significant differences were scored using the Student's t-test to calculate p-values.

### Cell culture and transient transfection assays

The HEK293 (HNF1B) cell line [45] contains a tetracycline-inducible HNF1B transgene. In a 96-well plate (17,500 cells/well) 30 ng of the promoter constructs were cotransfected using FuGeneHD (Roche) with 0.05 ng of renilla luciferase plasmid pRL-Con for normalization of transfection efficiencies. Four hours after transfection HNF1B expression was induced by the addition of 1 μg/ml doxycycline. Twenty-four hours after transfection firefly and renilla luciferase activities were measured in triplicate with the Dual-Luciferase Reporter Assay (Promega).

### Luciferase reporter assays in animal caps

50 pg reporter constructs and 150 pg HNF1B mRNA were coinjected into the animal region of Xenopus embryos at the two-cell stage together with renilla luciferase as internal control. Animal caps were cut, cultured in Steinberg's solution for four hours and pools of four caps were assayed by the Dual-Luciferase Reporter Assay (Promega). As the absolute levels of luciferase activity varied between pools of animal caps (data not shown, also described previously [42]), the Mann-Whitney-test was used to score significant differences.

## Authors' contributions

CD carried out all the experiments with the animal caps and the injected embryos and drafted the manuscript. SS made the transfection assays in cell culture. GUR conceived the study, participated in its design and coordination and helped to finish the manuscript. All authors read and approved the final manuscript.

## Supplementary Material

Additional file 1 **Table S1: Expression vectors with restriction enzymes and RNA polymerases used for RNA synthesis**. Click here for file

Additional file 2 **Table S2: List of primers used for quantitative RT-PCR**. Click here for file
