# Supplementary material for: The nephrogenic potential of the transcription factors osr1, osr2, hnf1b, lhx1 and pax8 assessed in Xenopus animal caps
Source: BMC Dev Biol. 2011 Jan 31;11:5. doi: 10.1186/1471-213X-11-5 (PMC3042965; doi:10.1186/1471-213X-11-5)
Supplement: Additional file 1 — Table S1: Expression vectors with restriction enzymes and RNA polymerases used for RNA synthesis. [file 1471-213X-11-5-S1.PDF]

Table S1:

	vector	restriction enzyme	RNA polymerase
**GFP**	pCS2+MT	PvuII	SP6
**OSR1**	pCS2+MT	NotI	SP6
**OSR2A**	pCMV-Tag2A	MluI	T3
**HNF1b**	myc-RC/CMV	SmaI	T7
**LHX1**	pCMV6 Entry	PinAI	T7
**PAX8**	pCMV6 Entry	PinAI	T7
