# Supplementary material for: The nephrogenic potential of the transcription factors osr1, osr2, hnf1b, lhx1 and pax8 assessed in Xenopus animal caps
Source: BMC Dev Biol. 2011 Jan 31;11:5. doi: 10.1186/1471-213X-11-5 (PMC3042965; doi:10.1186/1471-213X-11-5)
Supplement: Additional file 2 — Table S2: List of primers used for quantitative RT-PCR. [file 1471-213X-11-5-S2.PDF]

Table S2:

Primers produce amplicons with lengths of 90 to 124 bp.

gene symbol		sequence (5´→ 3´)
**anxa13**	for	CCAAAGGCAAGAAGTGAAGC
	rev	CTAATGCCGCCTTCTCAAAG
**c8a**	for	CTGCAGGATTTGATGGTCCT
	rev	TCTCTTTCCAGCTTGGCATT
**cer1**	for	ACCTTGCCCTTCACTCAGAA
	rev	TTGCTGATTTGGAACATGGA
**chrd**	for	GCTGTGGAGGAGAGTCGTTC
	rev	GCTGCAGGTTGAATCTAGCC
**cpn1**	for	GAAGAAGTGTGCAGGGAAGG
	rev	ACCGTGCATATTCCCAACAT
**esd**	for	ATCAAGCTGCCTCAGAGCAT
	rev	ACCAAAATCCCAGCTCTCCT
**fgfr4a**	for	AGTGAATGGAAGCCGATTTG
	rev	TCCGTAGGTGAAGCACCTCT
**fgfr4c**	for	AGACTCTGCAAACGCCAACT
	rev	TATGCGAATCAGTGGAGCAC
**gjb1**	for	GGGTCTGCATCGTTCTGAAT
	rev	TTGATGCTCTTTGCTGATGC
**gdnf**	for	TAAAGACCGAAAGCGGAAAA
	rev	TCAGTTCCTCCTTGGTTTCG
**gsc**	for	CAGCAATGCTCGTGTGTACC
	rev	CAAAGTGCCAACGTTCATGT
**hnf1a**	for	ACAGAAGAATCCCAGCAAGG
	rev	CTGAGCCTGTGATGGTGAGA
**hnf1b**	for	CAGTCCAGGGTTCTGGAAAT
	rev	CTGTCCTGGGACTGGATTTT
**hnf4a**	for	TATCCCGGCTTTCTGTGAAC
	rev	CTTTGTAGCCCCAAGAAGCA
**lhx1**	for	GTGCAACCTGACCGAGAAAT
	rev	GACTAGGTCACTGGGGGAGA
**ncor1**	for	AGCACAGCATGAAGGTGTTG
	rev	CCAATGGAACTTTGGTTGCT
**odc**	for	TGGGCTGGATCGTATCGTAGA
	rev	CATTGAATGTCGAGGCTGCA
**osr1**	for	ACCCTTCAAATGCCAGGAG
	rev	TTTGATTTTGGAAGGCTTGAG
**osr2**	for	TGCAGCTCACCAACTACTCC
	rev	ATGGTCTGTACAGCGCTCAG
**osr2A**	for	CATTCCAAAGAGAAACCATTCA
	rev	GGCATTTGTGGGGTGATT
**osr2B**	for	CATTCCAAAGAGAAACCATTCA
	rev	TGTAGGGCTTGATGTCTGC
**otx2**	for	TTTGCCAAAACTCGTTACCC
	rev	CACTTTGCTCTGCGGTTTTT

**pax2a**	for	GTGCTGCTCATGTCTTTCCA
	rev	TGGGTGGCTGTAAGGATTTC
**pax8**	for	TGGGATCCACGTATTCCATC
	rev	GCAGCTCTCCTGGTCACTGT
**pcdh8.2**	for	CTCGGGTGAAGTTCTGCTTC
	rev	GGAGTTTTGCCCTTCATCTG
**prodh2**	for	CAGTGCCCATTGAGGAAGAT
	rev	CGAGAGATCCACACAGTCCA
**rbms1**	for	AAGAGCCTGGCCTAGAGAGG
	rev	TGGGGTAAAACCCATTTTGA
**rpl35a**	for	TTGGAAAAAGATGTGCCTACG
	rev	CACCTTTCCCCAGATCACAC
**slc22a6**	for	TCTTGGTGGGACTAGCGTTC
	rev	TTCTGGGATCCACCATGAAT
**slc4a7**	for	TTGCGATATGTGCCTCTCTG
	rev	GGAACACAACAGCAGCTGAA
**slc5a2**	for	AGGCAGTGGGCATTTTGTAG
	rev	ACCAGGACCACAAACAAAGC
**slc7a8**	for	GAACCAGACATTGGCCTCAT
	rev	AGGCAATATTGGCAAAAACG
**tfe3**	for	CTAATGCTGCGCATACAGGA
	rev	GCGGTAGGCTCACTCTTCAG
**tmem27**	for	GGTTTGTCGTCACGTCTCCT
	rev	CGCTGTTAATCCGGTTTCTT
**trps1**	for	GCTATGTCTGCAATGCGTGT
	rev	TCTGCTCACCATTGTTCTGC
**ube3a**	for	TCTGGTGCAAGGACAACAAC
	rev	TTCCATGGCAATCATCTCAA
**wnt9b**	for	TCGATTCCTGCAGAACTTCC
	rev	GATTTAATGCCTGCGTTGCT
**wnt11**	for	TAACCTTTCTGCTGCACACG
	rev	CAGTGCTGGGTCTGATTCAA
**wnt11b**	for	TCCCCTGACTACTGCACAAA
	rev	GGTTGCAGCTGTCACTACCA
